# Hardware Removal and Conversion Hip Arthroplasty via a Single Interval Anterior Approach: Surgical Technique

**DOI:** 10.1016/j.artd.2024.101369

**Published:** 2024-04-17

**Authors:** Yaniv Steinfeld, Bheeshma Ravi, Daniel Pincus

**Affiliations:** a Division of Orthopaedic Surgery, Department of Surgery, University of Toronto, Toronto, Canada; b Division of Orthopaedic Surgery, Sunnybrook Health Sciences Centre, Toronto, Canada

**Keywords:** Revision total hip arthroplasty, Minimally invasive anterolateral approach, Conversion total hip arthroplasty, Anterior-based muscle-sparing (ABMS) approach, Direct anterior approach, Hardware removal

## Abstract

The supine ‘off-table’ anterior-based muscle-sparing (ABMS) approach is an established approach for primary total hip arthroplasty. The approach is performed with the patient positioned supine on a regular operating room table. It combines utilizing the Watson-Jones interval (without disrupting the abductor muscles) with principles of capsular management borrowed from the direct anterior approach. The approach may also be utilized for complex primary and revision hip arthroplasties. One clinical scenario the ABMS approach may be particularly well-suited to is conversion hip arthroplasty when retained hardware requires removal. The approach enables the surgeon to remove proximal femoral hardware and perform hip arthroplasty within the same muscle interval. This is in contrast to direct anterior approach, which entails separate windows being created on either side of the tensor fascia lata muscle to remove hardware and insert hip arthroplasty components, respectively. In this article, we describe our surgical technique for performing conversion total hip arthroplasty with hardware removal (sliding hip screw and plate in the discussed case) via a single interval with the supine off-table ABMS approach.

## Introduction

Orthopaedic surgeon and patient preferences regarding the approach for total hip arthroplasty (THA) have been shifting from the traditional “muscle-splitting” approaches (posterolateral [1] and direct lateral [2]) to “muscle-sparing” anterior approaches. The most popular among those is the direct anterior approach (“DAA”) [3]. The anterior-based muscle-sparing (ABMS) approach, [4] also known by several other names, [4] is another option for an anterior muscle-sparing approach.

THA utilizing the Watson-Jones interval was originally described by Röttinger and Bertin in 2004 [5] in the lateral decubitus position. Surgeons now also perform the approach with the patient positioned supine on a regular operating room (OR) table (ie, “off-table”). [6,7] One potential advance to the supine “off-table” technique compared to the original descriptions of Rottinger’s approach has been the combination of the Watson-Jones interval (without disrupting the abductor muscles) with principles of capsular management borrowed from the DAA. In practical terms, supine ABMS performed very similarly to the supine “off-table” DAA, but via the Watson-Jones interval lateral to the tensor fascia lata (TFL). As compared to the supine direct lateral approach, with supine ABMS, the superior capsule and obturator internus are released instead of the abductors to enable femoral access. There is now evidence in the literature supporting the use of this technique for primary THA for osteoarthritis [6,7] and femoral neck fracture. [8]

**Figure 1:**
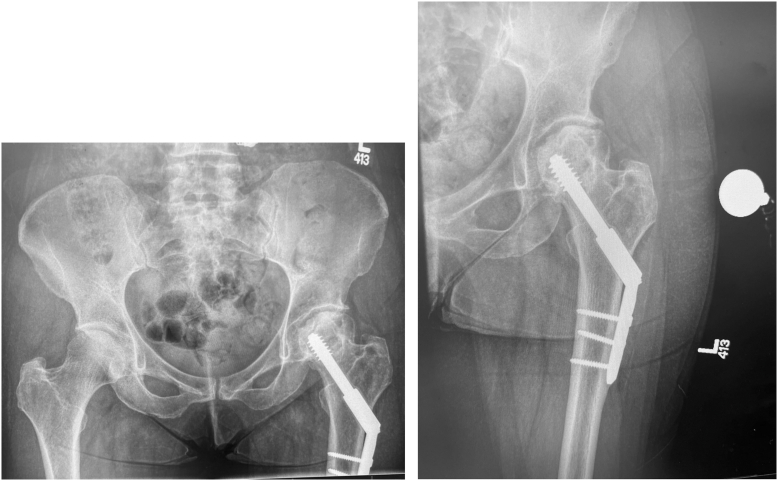
Preoperative anteroposterior pelvis and anteroposterior hip radiographs.

We have also utilized a supine “off-table” ABMS approach for complex primary and revision hip arthroplasties. One clinical scenario the ABMS approach may be particularly well-suited to is conversion hip arthroplasty when retained hardware requires removal. This is because the approach enables the surgeon to remove proximal femoral hardware and perform hip arthroplasty within the same muscle interval. In contrast, DAA generally entails creating separate windows on either side of the TFL to remove hardware and insert hip arthroplasty components, respectively. [9,10] In this article, we describe the technique and our experience with performing THA with hardware removal via a supine “off-table ABMS approach.” Figure 1 shows the preoperative anteroposterior pelvis and anteroposterior hip radiographs of an illustrative case: a 50-year-old female with avascular necrosis of the hip following femoral neck fracture fixation with a sliding hip screw and side plate.

### Surgical technique

In practical terms, supine ABMS is performed very similarly to the supine “off-table” DAA, but via the Watson-Jones interval lateral to the TFL. The patient is positioned supine on a regular OR table with a bump beneath the ipsilateral ischium to elevate the affected hip joint. Both legs are prepped and draped free. The groin region is sealed with adhesive drapes to mitigate its risk as a possible source of infection, which has been shown to be higher with anterior hip approaches. [11] The landmark for either a longitudinal or transverse (‘bikini’) incision is similar to the DAA but a centimeter or 2 more lateral. For a longitudinal incision, we try to palpate the TFL and make the incision on its lateral border. In the case of conversion THA with hardware removal, we prefer to incorporate the initial scar if possible. For example, in the case presented in Figure 1 there was an original incision for sliding hip screw insertion that was more distal than what would be optimal for THA. The long prior distal incision was utilized, which allowed for a larger mobile window for both hardware removal and hip reconstruction and allowed us to limit the extent of the proximal part of the incision. In general, at the proximal extent of the incision, we try to stop short of the hip crease to avoid wound breakdown in that area.

**Figure 2:**
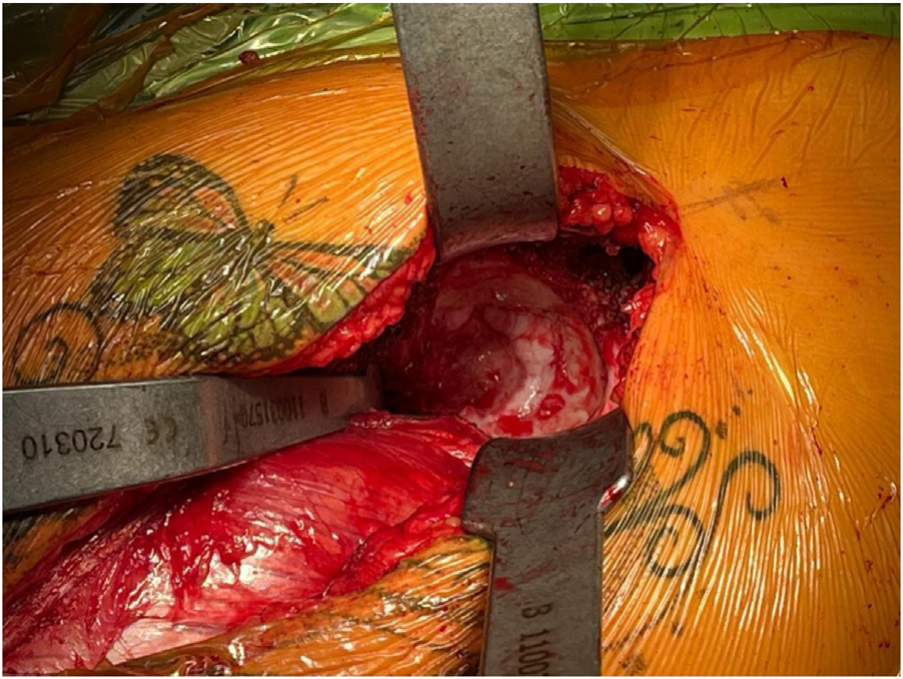
Acetabular exposure.

**Figure 3:**
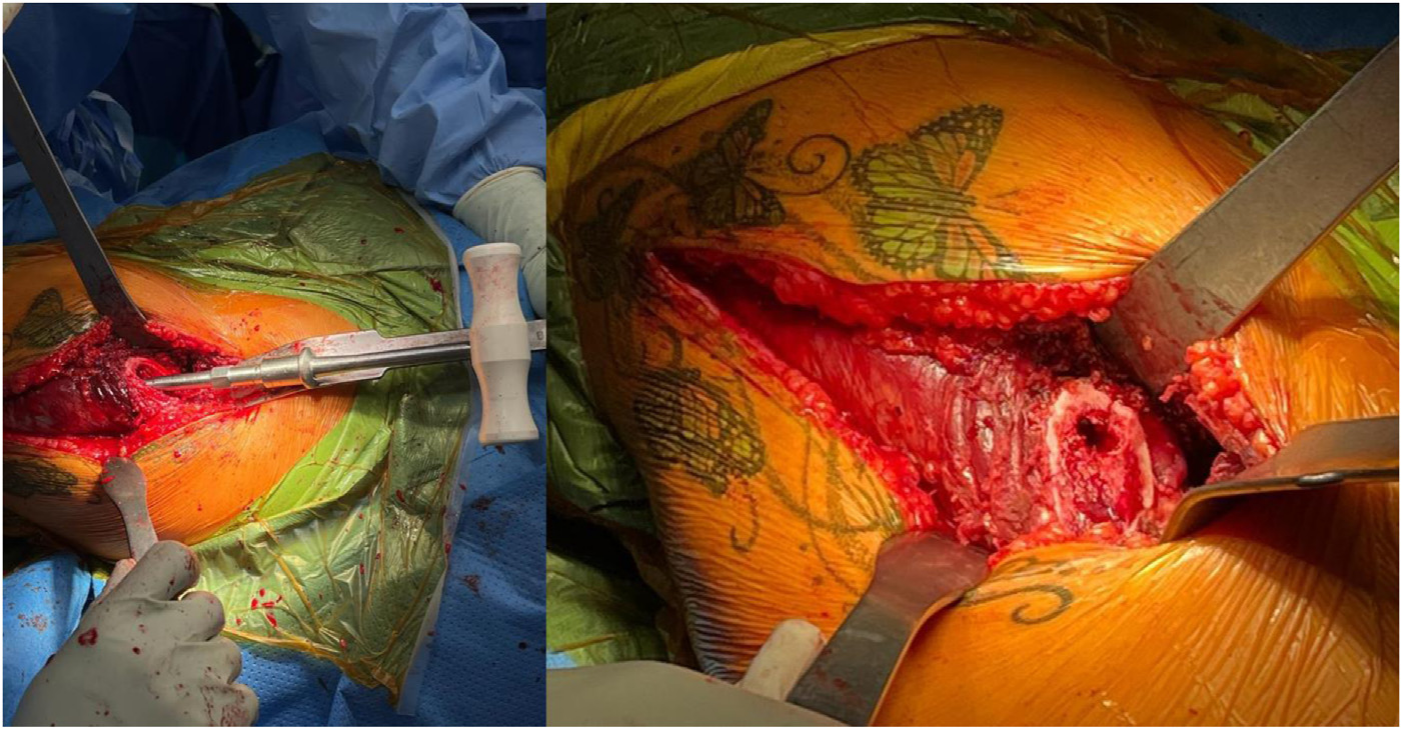
Femoral exposure.

The TFL-gluteus medius interval is developed bluntly, and small ‘gateway’/perforating veins are identified in this interval and can be coagulated. Lateral femoral circumflex vessels are not encountered and preserved. Capsulectomy or capsulotomy is performed. Distally, in this case, we performed a subvastus extension to identify and remove the existing hardware. At this point, the procedure continues in the same manner as a primary total hip replacement using the ABMS technique. [7] As previously mentioned in practical terms, this is very similar to the supine off-table DAA, but in the Watson-Jones interval lateral to the TFL. Figure 2 shows our typical acetabular exposure. Partial release of the indirect head of the rectus and inferior capsule are performed if needed for improved exposure. Following cup insertion, we prepare the femur by adducting and externally rotating the extremity with or without extension. The superior capsule and obturator internus are released (while protecting the gluteus medius and minimus) to enable femoral mobilization and elevation via a femoral elevator retractor behind the greater trochanter. Figure 3 demonstrates the femoral access that can be achieved with limited releases of the superior capsule and obturator internus and a femoral elevator behind the trochanter. We attempt to identify and preserve the insertions of the obturator externus and piriformis tendons in every case. We rely on anatomic referencing for component placement and do not use intraoperative fluoroscopy routinely, though it can readily be given the supine positioning.

**Figure 4:**
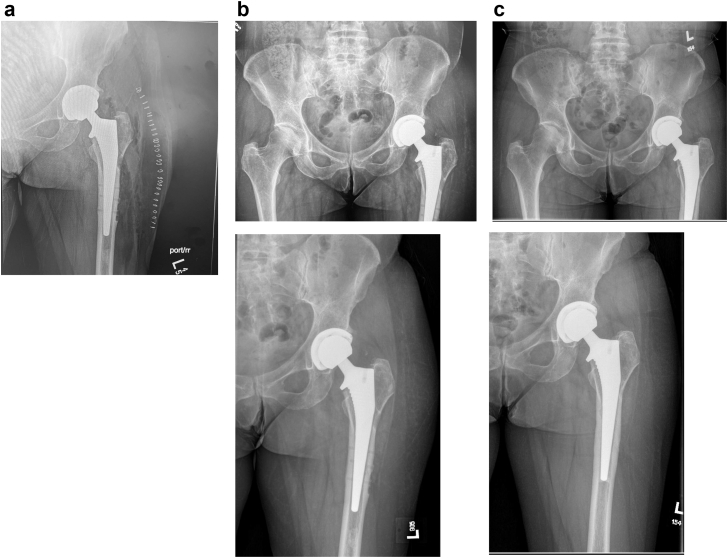
(a) Immediate postoperative radiograph; (b) 1-month postoperative radiograph; and (c) 9-month postoperative radiograph.

Figure 4 shows the initial, 1-month, and 9-month postoperative radiographs in this case. The patient has been followed thus far for 9 months postoperatively and continues to have excellent functional and radiographic results. We utilized a primary stem here as it was long enough to bypass the last screw hole from the removed sliding hip screw device. The cementless stem was chosen due to the patient’s young age (50s) and high prior activity level. We have also used cemented stems for conversion cases, as many of the patients requiring this procedure previously suffered a low-energy hip fracture and are by definition ‘osteoporotic’. Whether a cementless or cemented stem is chosen, our practice is generally to use a stem long enough to bypass the last screw hole from the removed hardware, ideally by 2 cortical diameters, to limit the theoretical risk of fracture propagation. [12] Postoperatively, the patient was managed weight-bearing as tolerated without specific hip precautions.

## Discussion

The supine “off-table” ABMS approach is an option for anterior THA that utilizes the Watson-Jones interval. It has been shown to be comparable to DAA in terms of clinical outcomes and complication rate. [13] Similar to the “off-table” DAA, supine positioning helps us evaluate leg length, stability, and soft tissue tension. It also facilitates intraoperative fluoroscopy when the surgeon believes it is helpful and/or indicated. As compared to DAA, the slightly more lateral skin incision and lateral fascial interval are further from the lateral femoral cutaneous nerve, which may help limit its neuropraxia. Another potential benefit may be that the lateral femoral circumflex vessels are not encountered and are more readily preserved. On the other hand, a potential disadvantage compared to the DAA is that though the approach is “muscle-sparing,” it is not “internervous.” Lower branches of the superior gluteal nerve to the TFL may be compromised.

As with all approaches, there is a learning curve for the surgeon not practicing this technique regularly, and we do not recommend performing conversion hip arthroplasty through this approach until mastery of the technique for primary THA is achieved. Similar to the DAA, femoral access via an ABMS approach is more challenging when encountering a varus/short neck, wide ilium, retroverted femur, obese, and/or muscular patient. In some of these scenarios, additional releases of the piriformis and obturator externus can help mobilize the femur, and femoral hyperextension via regular or traction OR tables can be utilized. Conversion THA with hardware removal via a single incision can also be performed using the more traditional posterolateral and direct lateral hip approaches. Here we have described a case of simultaneous hardware removal and hip arthroplasty, which is our preference. However, staging the 2 procedures is also an option when hardware removal is anticipated to be prolonged, challenging, and/or when significant bony defects are present.

## Summary

Of the different anterior hip approaches, a supine “off-table” ABMS technique may be particularly well-suited to conversion hip arthroplasty when retained hardware requires removal. We have found it to be an elegant option for these cases because the approach enables the surgeon to remove proximal femoral hardware and perform hip arthroplasty within the same incision and muscle interval.

## Conflicts of interest

The authors declare there are no conflicts of interest.

For full disclosure statements refer to https://doi.org/10.1016/j.artd.2024.101369.

## CRediT authorship contribution statement

**Yaniv Steinfeld:** Writing – original draft. **Bheeshma Ravi:** Writing – review & editing. **Daniel Pincus:** Writing – review & editing, Writing – original draft, Investigation, Conceptualization.

## Appendix A Supplementary Data

Conflict of Interest Statement for Ravi

Conflict of Interest Statement for Pincus

Conflict of Interest Statement for Steinfeld
